# Retraction: A Novel Copper Chelate Modulates Tumor Associated Macrophages to Promote Anti-Tumor Response of T Cells

**DOI:** 10.1371/journal.pone.0286555

**Published:** 2023-05-25

**Authors:** The PLOS ONE Editors

Following the publication and correction of this article [1, 2], concerns were raised results presented in Figs 3, 5, and 6. Specifically,

- The following Untreated Control RT-PCR results appear similar:
  ○ Fig 3A IFN-γ and Fig 5A IFN-γ;
  ○ Fig 3A IL-4 and Fig 5A IL-4;
  ○ Fig 3A TGF-β and Fig 5A TGF-β;
  ○ Fig 3A GAPDH and Fig 5A GAPDH.
- The following FACS results appear more similar than would be expected from independent results:
  ○ The lower left and right quadrants of the Fig 5D IFN-γ “Untreated unfixed TAMs” and TGF-β “CuNG-treated unfixed TAMs, in transwell” panels;
  ○ The Fig 6C 72h “In vitro CuNG Treated TAMs” panel and 96h “In vitro CuNG Treated TAMs panel”.

Although the similar untreated control samples appear to represent the same experimental conditions, the authors commented that the Fig 3A results were obtained from experiments conducted at a different time than the Fig 5A results, calling into question whether one or both of these experiments were conducted alongside the appropriate controls. The corresponding author provided gel data underlying the published Fig 3A results; these underlying data were incomplete and suggest that the Fig 3A results were prepared from spliced gels. The original data underlying Fig 5A are no longer available.

Additional concerns were raised regarding data presented in Figs 3A and 5A, but due to the low resolution of the published figures and the incomplete underlying data set, the journal has not been able to assess the merit of the additional concerns raised with these figures.

The authors explained that the Fig 5D experiments were conducted on the same day and on the same instrument. However, this does not clarify the level of similarity observed between these two panels. Regarding the similarity between the Fig 6C results, the authors commented that it is possible an error was made during the figure preparation. The authors provided the data underlying the Fig 6C results and a replacement Fig 6. However, the original FACS data underlying Fig 5D are no longer available. In the absence of the original FACS data underlying Fig 5D, the journal is unable to confirm the FACS results presented in this figure.

In light of the concerns affecting multiple figure panels that question the reliability of these data, the *PLOS ONE* Editors retract this article.

SG agreed with the retraction. SKC and SC did not agree with the retraction. AM, JMB, PC, AG, AA, DM, RB, MA, JB, PKD, GS, MC, and TD either did not respond directly or could not be reached.
